# Measurement properties of movement smoothness metrics for upper limb reaching movements in people with moderate to severe subacute stroke

**DOI:** 10.1186/s12984-024-01382-1

**Published:** 2024-05-29

**Authors:** Gwenaël Cornec, Mathieu Lempereur, Johanne Mensah-Gourmel, Johanna Robertson, Ludovic Miramand, Beatrice Medee, Soline Bellaiche, Raphael Gross, Jean-Michel Gracies, Olivier Remy-Neris, Nicolas Bayle

**Affiliations:** 1 Department of Physical and Rehabilitation Medicine, CHU Brest, Brest, F-29200 France; 2 UMR 1101 LaTIM, Univ Brest, INSERM, Brest, F-29200 France; 3 Pediatric Physical and Rehabilitation Medicine Department, Fondation Ildys, Rue Alain Colas, Brest, F-29200 France; 4 Physical Medicine and Rehabilitation Department, AP-HP, Raymond Poincaré Hospital, Université Paris-Saclay, Team INSERM 1179, UFR de Santé Simone Veil, Versailles Saint-Quentin university, Garches, France; 5 Department of Neurological Physical Medicine and Rehabilitation, Henry-Gabrielle hospital, Hospices Civils de Lyon, Saint-Genis-Laval, France; 6 Nantes Université, CHU Nantes, Movement - Interactions - Performance, MIP, UR 4334, Nantes, F-44000 France; 7 Service de Rééducation Neurolocomotrice, Unité de Neurorééducation, AP-HP, Hôpitaux Universitaires Henri Mondor, Créteil, F-94010 France; 8 Laboratoire Analyse et Restauration du Mouvement, UR 7377 BIOTN, Université Paris Est Créteil (UPEC), Créteil, France

**Keywords:** Measurement properties, Reaching, Kinematics, Smoothness, Stroke

## Abstract

**Background:**

Movement smoothness is a potential kinematic biomarker of upper extremity (UE) movement quality and recovery after stroke; however, the measurement properties of available smoothness metrics have been poorly assessed in this group. We aimed to measure the reliability, responsiveness and construct validity of several smoothness metrics.

**Methods:**

This ancillary study of the REM-AVC trial included 31 participants with hemiparesis in the subacute phase of stroke (median time since stroke: 38 days). Assessments performed at inclusion (Day 0, D0) and at the end of a rehabilitation program (Day 30, D30) included the UE Fugl Meyer Assessment (UE-FMA), the Action Research Arm Test (ARAT), and 3D motion analysis of the UE during three reach-to-point movements at a self-selected speed to a target located in front at shoulder height and at 90% of arm length. Four smoothness metrics were computed: a frequency domain smoothness metric, spectral arc length metric (SPARC); and three temporal domain smoothness metrics (TDSM): log dimensionless jerk (LDLJ); number of submovements (nSUB); and normalized average rectified jerk (NARJ).

**Results:**

At D30, large clinical and kinematic improvements were observed. Only SPARC and LDLJ had an excellent reliability (intra-class correlation > 0.9) and a low measurement error (coefficient of variation < 10%). SPARC was responsive to changes in movement straightness (r_Spearman_=0.64) and to a lesser extent to changes in movement duration (r_Spearman_=0.51) while TDSM were very responsive to changes in movement duration (r_Spearman_>0.8) and not to changes in movement straightness (non-significant correlations). Most construct validity hypotheses tested were verified except for TDSM with low correlations with clinical metrics at D0 (r_Spearman_<0.5), ensuing low predictive validity with clinical metrics at D30 (non-significant correlations).

**Conclusions:**

Responsiveness and construct validity of TDSM were hindered by movement duration and/or noise-sensitivity. Based on the present results and concordant literature, we recommend using SPARC rather than TDSM in reaching movements of uncontrolled duration in individuals with spastic paresis after stroke.

**Trial Registration:**

NCT01383512, https://clinicaltrials.gov/, June 27, 2011.

**Supplementary Information:**

The online version contains supplementary material available at 10.1186/s12984-024-01382-1.

## Highlights

Reliability, responsiveness and construct validity of SPARC were satisfactory.

Responsiveness and construct validity of LDLJ, NARJ and nSUB were highly related to movement duration.

LDLJ had an excellent reliability and a low measurement error, but not NARJ and nSUB.

**Supplementary Information:**

The online version contains supplementary material available at 10.1186/s12984-024-01382-1.

## Introduction

Spastic paresis of the upper extremity (UE) was reported in 48% of survivors at 1 week after stroke in a community-based population (*n* = 421), with full UE function achieved at discharge by 79% of those with mild paresis but only 18% of those with severe paresis [1]. Three main symptoms are well described in spastic paresis syndrome [2]: structural alterations relating to immobility (spastic myopathy, leading to muscle contractures) [3, 4], impaired motor control (stretch-sensitive paresis) of the agonist muscles [5, 6], and overactivity of antagonist muscles [7, 8], (including spasticity [8–10], spastic dystonia [11] and spastic cocontractions [12–15]).

Spastic paresis directly alters the movement trajectories and velocity with spatial (poor movement control, less efficient trajectories) and temporal (longer movement duration) discontinuities, resulting in a lack of smoothness [16–18]. Changes in the smoothness of the hand trajectory after stroke have been studied during reaching, grasping, and pointing movements [19], and the evaluation of smoothness has been suggested as a valid indicator of the quality of spontaneous motor recovery [20–23] and rehabilitation-induced recovery [18, 24–26].

The assessment of measurement properties of smoothness metrics is needed for the evaluation of changes in the poststroke spastic paretic UE. To date, many metrics have been used to explore movement recovery after stroke [27]. Research involving robotic rehabilitation systems in the last fifteen years has particularly contributed to the development of kinematic metrics, including smoothness, as potential biomarkers for movement recovery [24, 25, 27–29]. However, the use of smoothness metrics in clinical research remains limited, as those metrics require particular instrumentation and expertise that might be an obstacle for multicentric studies, are often insufficiently defined mathematically (some are even robot-specific metrics) and validated, and are often non-reproducible, non-dimensionless (i.e. highly relying on movement time), poorly robust against measurement noise, or are not related to the intermittency of movement [19, 27, 30].

New smoothness metrics that attempt to avoid those limitations have been developed and used to assess point-to-reach and point-to-grasp movement in healthy subjects and individuals after stroke [23, 31–33], namely the log dimensionless jerk (LDLJ), a smoothness metric conceived in the temporal domain and the spectral arc length metric (SPARC). The SPARC was conceived in the frequency domain by Balasubramanian and colleagues, notably to overcome the bias of movement duration and noise-sensitivity in previously developed smoothness metrics, who tested its content validity and described it as a robust to noise, sensitive, reliable, and practical metric after tests on mathematical models [30, 34].

In an earlier study, we compared the properties of four smoothness metrics currently used in the literature (SPARC, and three temporal domain smoothness metrics (TDSM): LDLJ, number of zero-crossings in the acceleration profile also called number of submovements (nSUB) and normalized average rectified jerk (NARJ)) during UE reaching movements in 32 middle-aged healthy participants [33]. In this setting, the SPARC had the lowest measurement error, and seemed independent of movement duration whereas the TDSM were highly time-dependent. A better understanding of the measurement properties of these metrics is still needed for patients with poststroke UE impairment. An international consensus was reached on the taxonomy, terminology and definitions of measurement properties within the COSMIN initiative (COnsensus-based Standards for the selection of health Measurement INstruments) setting a framework for the present study [35].

This study aimed to assess the measurement properties (reliability, responsiveness and construct validity) of the SPARC and three TDSM (NARJ, LDLJ and nSUB) for point-to-reach movements in people with moderate to severe impairment in the subacute phase of stroke, before and after a rehabilitation program.

Based on our previous work in healthy subjects [33] and literature, we hypothesized that the three TDSM would be more associated with movement duration while the SPARC would be more associated with movement straightness in the present context.

## Methods

This was an ancillary study of the REM-AVC (*Ré-Éducation Mécanisée après Accident Vasculaire Cérébral* – Mechanized rehabilitation after cerebrovascular accident) multicenter single-blinded prospective randomized controlled trial, which compared the effects of 20 days (4 weeks, 5 days a week) of self-rehabilitation using a mechanized device with control self-exercises on UE impairment in people in the subacute phase of stroke. More details can be found in the original publication of the study [36]. It was conducted in accordance with the Declaration of Helsinki, Good Clinical Practice guidelines and local regulatory requirements (registration number, ID-RCB: NCT01383512, https://clinicaltrials.gov/, registered June 27, 2011), and was approved by the Brest University Hospital Institutional Review Board (n°653). All participants gave written consent to the use of their data.

### Sample

Of the 218 individuals included in the REM-AVC trial, 37 participants in three centers underwent motion capture of their paretic UE. Six participants were excluded: two did not complete both motion capture assessments and four had uninterpretable data (many artefacts). Among the 31 included, the median (Q1 – Q3) age was 64 (54–72) years and 22 (71%) were males. Twenty-three (74%) participants had experienced an ischemic stroke, 8 a hemorrhagic stroke in the middle cerebral artery territory and the spastic paresis syndrome affected the dominant side in 14 (45%) participants. The median (Q1 – Q3) initial NIHSS score was 11 (7.5–15.5) points and the median (Q1 – Q3) time since stroke was 38 (25–62) days.

### Clinical assessments

The clinical metrics were the upper extremity Fugl-Meyer assessment (UE-FMA, ranging from 0 to 66), the Action Research Arm Test (ARAT, ranging from 0 to 57), a composite Modified Ashworth Scale (cMAS – the sum of the scores of the elbow flexors and extensors, wrist and finger flexors) and the shoulder passive range of motion (PROM). Each outcome was assessed twice: at inclusion (Day 0, D0) and at the end of the rehabilitation protocol (Day 30, D30). All assessments were performed by a blinded investigator. The proximal subscore of UE-FMA (maximum score: 42) was secondarily calculated by excluding from the original score the hand and wrist (on 24 points) assessments as they are less involved in the smoothness of reaching movements.

### Experimental set-up

**Fig. 1:**
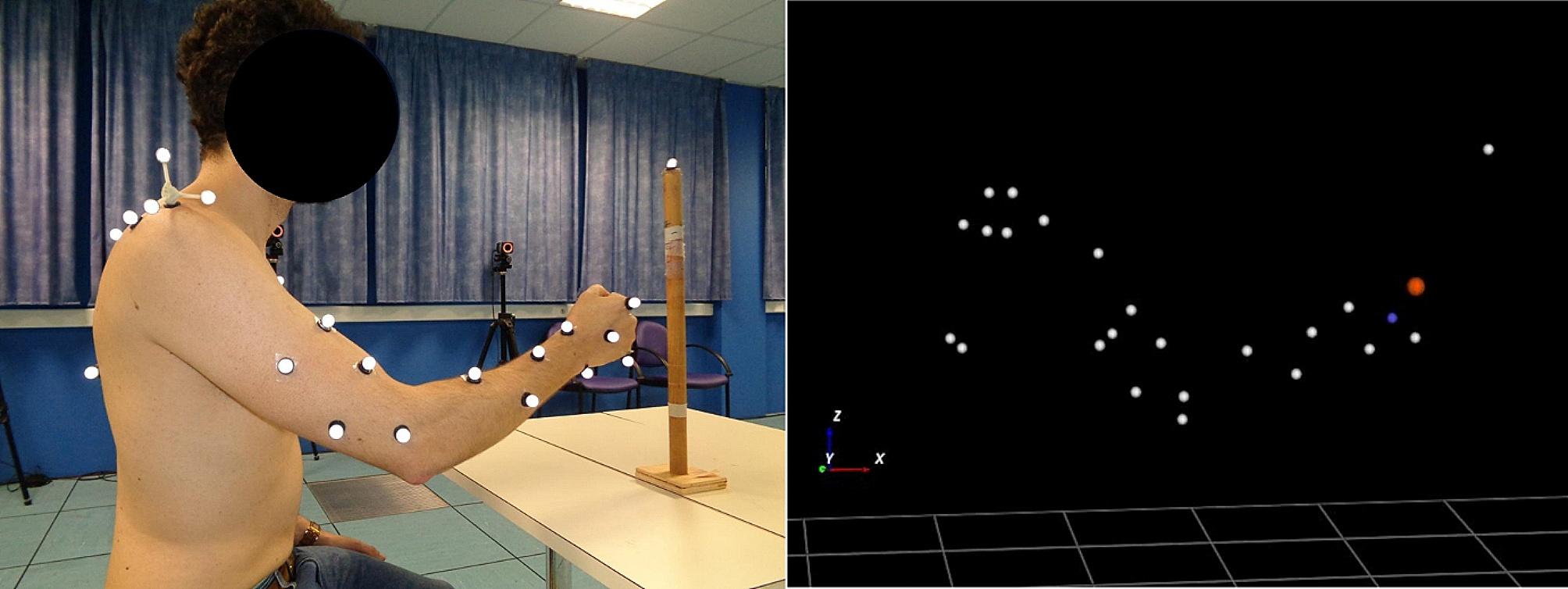
Marker placement (left) and 3D reconstruction (right) during motion analysis

Participants underwent two 3D motion analysis sessions at D0 and D30, during which they performed a reaching task (i.e. reach-to-point) with the impaired UE. Twenty-five reflective markers (14 mm) were placed on UE and trunk anatomical landmarks, by the same investigator at each session, following the International Society of Biomechanics recommendations [37] as illustrated in Fig. 1. Marker trajectories were recorded using a six, eight or nine camera motion capture system (Vicon, MX13 and FX20 camera models, Oxford, UK) at 120 Hz.

### Blue: mid-hand marker; red: head of the second metacarpal marker

**Fig. 2:**
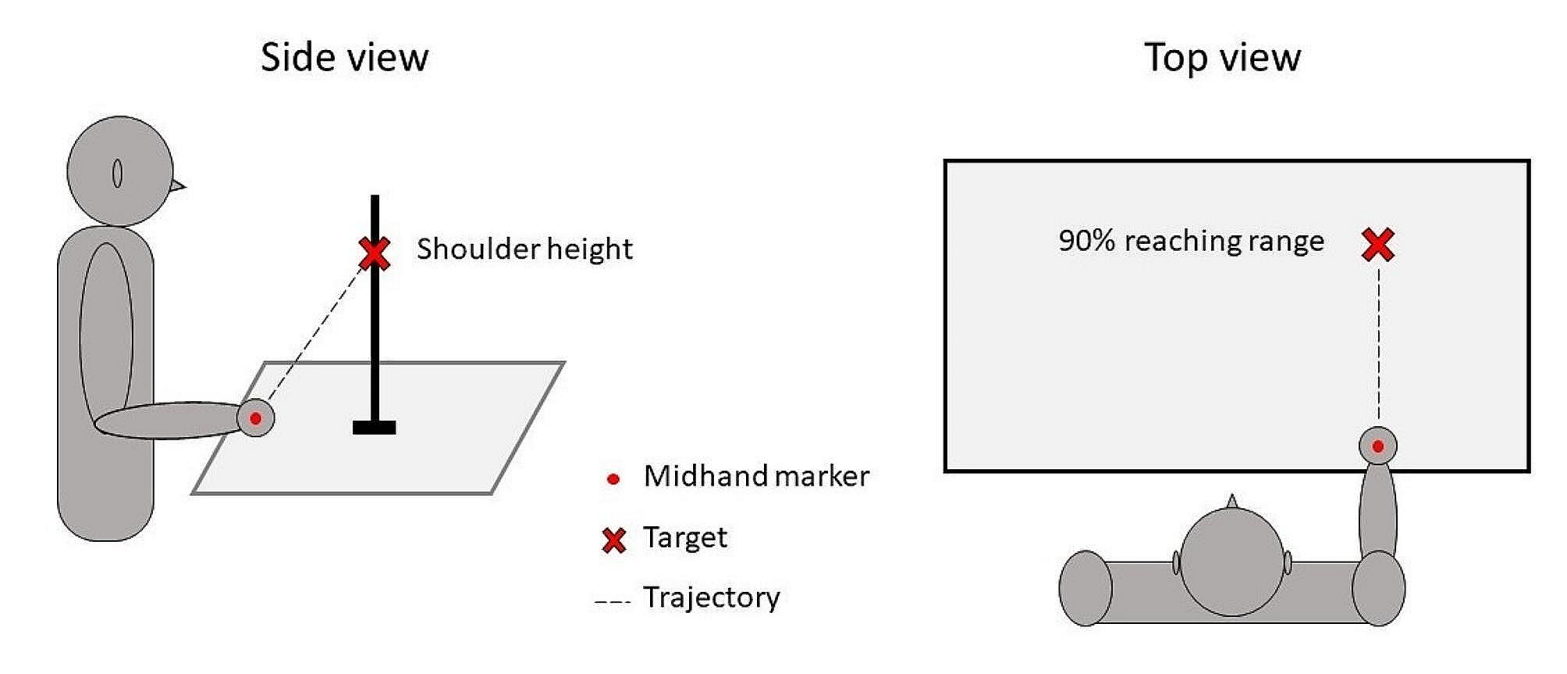
Representation of the motion analysis set-up at the starting position

Participants were seated with their closed fist resting on a table and unconstrained trunk. The shoulder was at 0 degrees of flexion and abduction, and the elbow was flexed at 90 degrees in a neutral pronation-supination position. Participants were asked to reach with their closed fist, at comfortable speed, as close as possible to a single target indicated by a mark on a vertical stick and located in front of them, at 90% of the length of their upper limb and at the clavicle level. The set-up is represented in Fig. 2. The movement was repeated four times, the first attempt being considered as a training movement and thus not recorded. Thus for all participants, a total of 93 movements were recorded and analyzed at each session.

### Data analysis

The analyses presented here are focused on the mid-hand marker (placed over the middle of the third metacarpal bone, on the back of the hand). Each recorded trajectory was visually inspected twice by the same investigator to manually define the beginning and end of movements. The beginning of the movement was defined as the first ascending point of the trajectory in an upward direction. The end of the movement was the furthest point of the trajectory in the anteroposterior direction. If large artefacts were observed, the mid-hand marker was replaced by the marker placed over the head of the second metacarpal. Marker position data were computed using WorkStation 5.2.9 (Oxford Metrics, Oxford, UK).

A second order, zero-lag, low-pass Butterworth filter with a 6 Hz cutoff frequency was applied to the trajectories using Python [38] before analyses, except for the SPARC as recommended by its authors [30] because it has an in-built filter. The 6 Hz cut-off frequency was based on previous work studying the effect of filtering on TDSM [31]. The mean value of the three movements was used in the analyses for each outcome. Python was used for all calculations. First, second, and third derivatives of 3D trajectory of the mid-hand marker data were calculated to retrieve the velocity, acceleration, and jerk profiles. Peak velocity and peak acceleration were recorded.

Smoothness was quantified using the SPARC (with V_threshold_ = 0.05 and ω_c_^max^ = 20 Hz as recommended by Balasubramanian et al. [30]) and three temporal domain smoothness metrics (TDSM): NARJ, LDLJ, and nSUB. SPARC and LDLJ are negative metrics (an increase in magnitude towards 0 indicates increased smoothness) whereas NARJ and nSUB are positive metrics (an increase in magnitude indicates a reduction in smoothness). A mathematical description of the metrics is available in a prior publication [33].

The index of curvature (IoC), a measure of movement straightness defined as the ratio of the arc length of the trajectory to the length of the straight line linking the first and the last movement points [39] was calculated. It is reported to approximate movement efficiency in the case of pathological movement [40]. The Python code provided by Balasubramanian et al. [30] for computing SPARC and LDLJ was edited to include the calculation of all the kinematic metrics.

### COSMIN measurement properties

#### Reliability and measurement error

Reliability is the degree to which the instrument is free from measurement error. Measurement error is the error of a patient’s score that is not attributed to true changes in the construct. Participants could have a short break between tries. The investigator in charge of 3D motion analysis recording and treatment did not know the value of the kinematic or clinical metrics between tries or between sessions as all calculations were made after the end of the study.

#### Responsiveness

Responsiveness is the extent to which an instrument truly measures change, by comparing changes in an instrument of interest with changes in a gold standard. No gold standard is established in smoothness metrics; thus, we chose to measure changes in smoothness measures between D0 and D30 as compared with changes in related constructs. Movement smoothness is defined as a quality related to non-intermittency, and lack of movement smoothness can be explained by arrests or deviations in its trajectory, independently of its amplitude and duration [30]. Direct consequences of a lack of smoothness can be an increase in the spatial and temporal components of the movement. We hypothesized that changes in a metric measuring movement smoothness would be correlated with changes in IoC, and, to a lesser extent, with changes in movement duration because IoC, as a measure of trajectory (position as a function of time); explores both the spatial and temporal components of movement. As discussed by COSMIN experts and Angst [41], effect size is not a proper measure of responsiveness but it is still a complementary measure if previous hypotheses are made, in the present case that smoothness metrics should display an effect size closer to IoC than to movement duration.

#### Construct validity

Hypotheses-testing validity is the degree to which the scores of an instrument at one moment in time are consistent with hypotheses. We hypothesized that:

- Smoothness metrics would be positively correlated with each other, with kinematic (IoC, movement duration) and clinical metrics (UE-FMA, UE-FMAp, ARAT) at D0 and at D30.
- Smoothness metrics at D0 would be positively correlated with kinematic (IoC, movement duration) and clinical metrics (UE-FMA, UE-FMAp, ARAT) at D30 (predictive validity).
- Smoothness metrics absolute values would be lower for LDLJ and SPARC, and higher for NARJ and nSUB, than those published in healthy subjects [33].

Criterion validity is the degree to which the scores of an instrument are an adequate reflection of a “gold standard”. No gold standard is available for smoothness metrics in the present context; thus, criterion validity was not assessed.

### Statistics

As participants in both REM-AVC groups received the same amount of treatment and as no demographic, clinical or kinematic variables differed, participants were pooled into a single sample for the purpose of this study. Descriptive statistics were performed to calculate the median values and interquartile intervals.

The normality of data was assessed by visual inspection of the data distribution and a Shapiro‒Wilk test. As most data had a non-normal distribution due to ceiling effects and sample size, only nonparametric tests were used. Comparisons between D0 and D30 clinical and kinematic variables were performed using Wilcoxon tests, and effect sizes were calculated ([0.1–0.3[: small; [0.3–0.5[: medium; ≥0.5: large). A figure displaying mean and standard deviation of trajectory and velocity profiles was made for visual analysis.

#### Reliability and measurement error

Reliability of smoothness metrics was assessed by intra-class correlation (ICC) estimates and their 95% confident intervals (2 ways mixed effects, average measures (k = 3), absolute agreement). ICC values less than 0.5, between 0.5 and 0.75, between 0.75 and 0.9, and greater than 0.90 were interpreted as indicative of poor, moderate, good, and excellent reliability, respectively [42]. Measurement error was assessed by the median of intra-individual coefficients of variation (CoV, which is the ratio of the standard deviation to the mean of the three recorded tries) at D0 and at D30.

#### Responsiveness

Changes between D0 and D30 (Δ) were calculated as follow: Δ = D30 value – D0 value. Responsiveness was assessed with Spearman correlations between changes in smoothness metrics and changes in IoC and movement duration.

#### Construct validity

Hypotheses were tested using Spearman correlations between smoothness metrics and clinical metrics (UE-FMA, UE-FMAp and ARAT) and kinematic metrics (movement duration and IoC) at D0 and at D30; and between smoothness metrics at D0 and clinical and kinematic metrics at D30.

Spearman’s r was interpreted as weak if < 0.4, moderate if [0.4–0.6[, strong if [0.6–0.8[ and very strong if ≥ 0.8. All statistical analyses were performed using SPSS v20 (IBM, Armonk, NY).

## Results

The only missing demographic, clinical or kinematic data was shoulder range of motion at D0 for one participant. Database for main metrics is available in Appendix A.

### Clinical changes

Changes in clinical metrics are presented in Table 1. The UE-FMA total and proximal subscore and ARAT scores were improved at D30 (effect size: large). cMAS and shoulder PROM did not change between D0 and D30.

**Table 1:** Baseline and final clinical metrics and comparison

Clinical measures	Day 0		Day 30		Difference	Effect size
	Median	Q1 – Q3	Median	Q1 – Q3	*p*-value	
UE-FMA	27	19–33	45	33–52	< 0.0001	0.87
UE-FMA: proximal subscore	24	17–27	37	26–42	< 0.0001	0.86
ARAT	10	3–19	31	19–45	< 0.0001	0.85
cMAS	3	1–4	3	1–4	0.4	*-*
Shoulder PROM						
Anterior flexion	150	110–170	150	120–170	0.3	*-*
Abduction	103	90–153	105	90–160	0.8	*-*
External rotation	40	15–55	40	20–60	0.2	*-*

Q: quartile, UE-FMA: upper extremity Fugl Meyer assessment, cMAS: composite modified Ashworth scale, ARAT: Action Research Arm Test, PROM: passive range of motion

### Kinematic changes

**Fig. 3:**
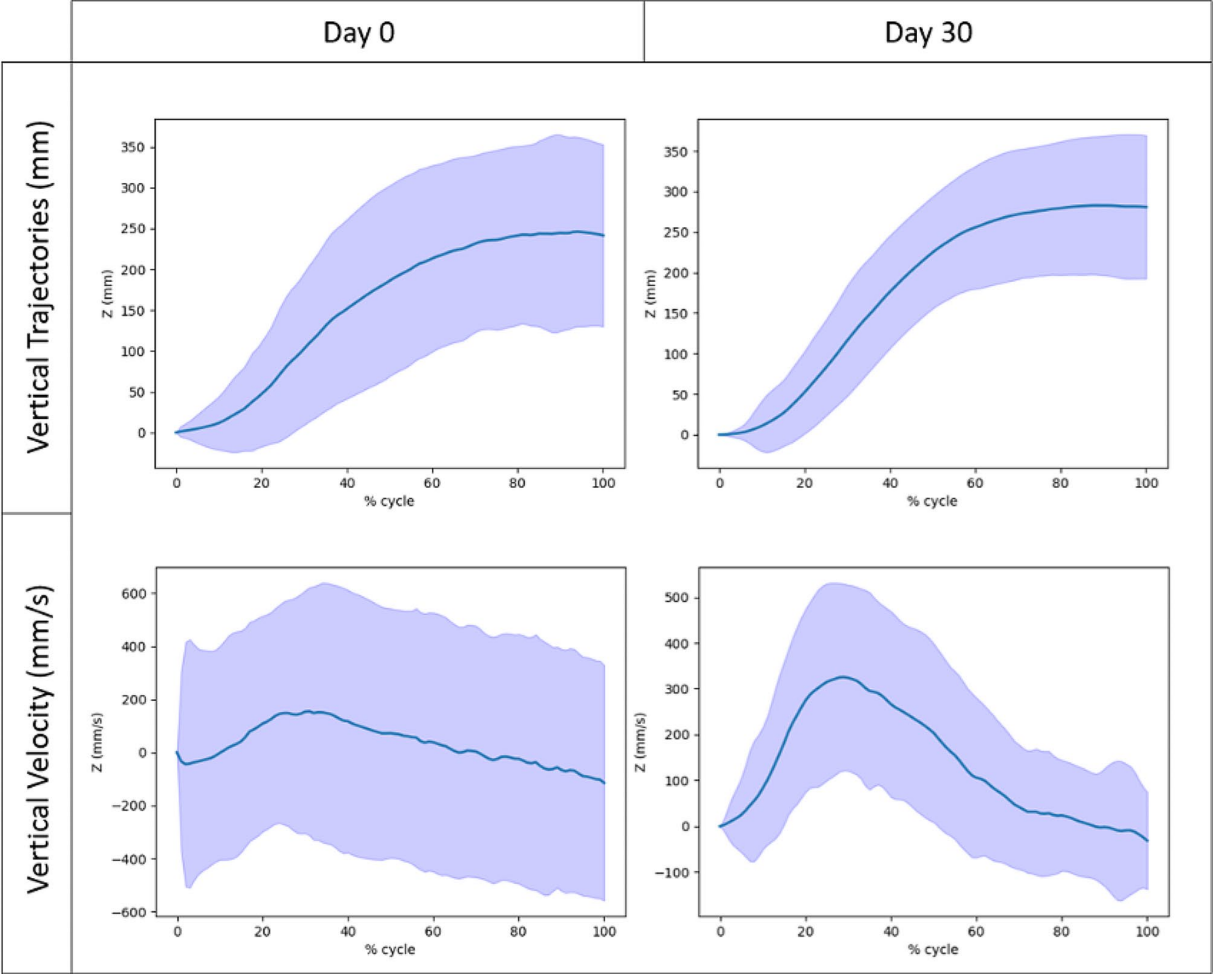
Trajectories and velocity profiles of day 0 (D0) and day 30 (D30) reaching movements. Blue line: mean value, lavender: standard deviation area

Changes in kinematic metrics are presented in Table 2. Trajectories and velocity profiles visually improved between D0 and D30 as illustrated in Fig. 3. Movements at D30 were significantly shorter in duration and trajectory (less distance covered to reach the target), straighter, faster and smoother according to all four smoothness metrics (medium (mean velocity) to large effect sizes). Peak velocity and peak acceleration did not change significantly.

**Table 2:** Baseline and final kinematic metrics and comparison

Kinematic metrics	D0		D30		Difference	Effect size
	Median	Q1 – Q3	Median	Q1 – Q3	*p*-value	
Duration (s)	2.6	2.1–3.9	2	1.7–2.6	0.0003	0.64
*CoV*_*intra*_	*16*	*11–25*	*13*	*7–24*	*0.9*	-
Trajectory length (mm)	552	461–709	437	406–623	0.001	0.58
*CoV*_*intra*_	*7*	*4–13*	*4*	*3–9*	*0.9*	*-*
IoC (%)	35.2	22.9–79.2	13.8	10.7–23.1	< 0.0001	0.80
*CoV*_*intra*_	*25*	*19–48*	*35*	*17–45*	*0.8*	*-*
Mean velocity (mm/s)	213	127–273	246	185–319	0.02	0.43
*CoV*_*intra*_	*13*	*10–21*	*13*	*7–20*	*0.4*	*-*
Peak velocity (mm/s)	600	391–721	682	480–745	0.4	*-*
*CoV*_*intra*_	*12*	*8–20*	*11*	*7–15*	*0.06*	*-*
Peak acceleration (mm/s²)	2768	1860–3650	3169	1899–4523	0.4	*-*
*CoV*_*intra*_	*23*	*11–36*	*21*	*13–37*	*0.5*	*-*
SPARC	-1.82	-2.14 – -1.70	-1.61	-1.78 – -1.51	< 0.0001	0.76
*CoV*_*intra*_	*8.9*	*6–14*	*4.1*	*2–10*	*0.03*	*0.39*
LDLJ	-10.7	-12.7 – -9.83	-9.36	-11,0 – -8.05	0.0003	0.65
*CoV*_*intra*_	*9.1*	*6–13*	*7.8*	*4–13*	*0.2*	*-*
nSUB	15	12–28	11	7–17	0.0009	0.59
*CoV*_*intra*_	*33*	*22–46*	*32*	*20–43*	*0.9*	*-*
NARJ 10^− 5^ (mm/s³)	3.8	1.88–9.71	1.52	0.79–3.31	0.0003	0.65
*CoV*_*intra*_	*48*	*28–75*	*38*	*19–57*	*0.2*	*-*

Q: quartile, CoV_intra_: intra-individual coefficient of variation, SPARC: spectral arc length metric, LDLJ: log dimensionless jerk, nSUB: number of submovements, NARJ: normalized average rectified jerk

### Reliability and measurement error

SPARC and LDLJ had close ICC estimates (0.912 [0.861;0.946] and 0.911 [0.861;0.945] respectively) indicating excellent reliability, followed by nSUB (0.891 [0.830;0.933]) indicating good reliability and NARJ (0.613 [0.391;0.763]) indicating moderate reliability. Among the smoothness metrics, SPARC had the smallest CoV both at D0 and at D30 and was the only metric for which CoV was significantly improved at D30 (medium effect size). CoV at D0 and at D30 were also less than 10% for LDLJ but was greater than 30% for NARJ and nSUB.

### Responsiveness

Correlations between the changes in kinematic metrics between D0 and D30 are presented in Table 3. ΔSPARC was moderately correlated with the changes in movement duration and strongly correlated with ΔIoC. ΔTDSM were very strongly correlated with movement duration, but not with ΔIoC. ΔSPARC and ΔTDSM were moderately to strongly correlated. ΔTDSM were strongly to very strongly correlated with each other. Effect size of change between D0 and D30 for the SPARC (0.76) was closer to the one of IoC (0.80), while it was closer to the one of movement duration (0.64) for those of LDLJ (0.65), NARJ (0.65) and nSUB (0.59).

**Table 3:** Spearman (r) correlations between changes in metrics from day 0 (D0) to day 30 (D30)

	ΔDuration	ΔIoC	ΔnSUB	ΔNARJ	ΔLDLJ
ΔSPARC	-0.51^**^	-0.64^**^	-0.54^**^	-0.59^**^	0.67^**^
ΔLDLJ	-0.81^**^	-0.26	-0.84^**^	-0.73^**^	
ΔNARJ	0.90^**^	0.35	0.89^**^		
ΔnSUB	0.96^**^	0.33			

^**^: *p* < 0.01, Δ: D30-D0 value, IoC: index of curvature, SPARC: spectral arc length metric, LDLJ: log dimensionless jerk, nSUB: number of submovements, NARJ: normalized average rectified jerk

### Construct validity

1. At D0.

SPARC was moderately correlated with UE-FMA and its proximal subscore and strongly correlated with ARAT. TDSM were weakly or insignificantly correlated with UE-FMA and moderately correlated with ARAT. SPARC was very strongly correlated with IoC and moderately correlated with movement duration; TDSM were moderately correlated with IoC and very strongly correlated with movement duration. Overall results at D0 are presented in Table 4.

**Table 4:** Spearman correlations (r) between metrics at day 0

	UE-FMA	UE-FMAp	ARAT	Duration	IoC
SPARC	0.48^**^	0.56^**^	0.68^**^	-0.59^**^	-0.87^**^
LDLJ	0.39^*^	0.28	0.46^**^	-0.89^**^	-0.57^**^
NARJ	-0.36^*^	-0.23	-0.46^**^	0.87^**^	0.56^**^
nSUB	-0.30	-0.16	-0.39^*^	0.93^**^	0.41^*^

^*^: *p* < *0,05*, ^**^: *p* < 0.01, UE-FMA: upper limb Fugl Meyer score, UE-FMAp: proximal subscore of UE-FMA, ARAT: Action Research Arm Test, IoC: index of curvature, SPARC: spectral arc length metric, LDLJ: log dimensionless jerk, nSUB: number of submovements, NARJ: normalized average rectified jerk

2) At D30.

All smoothness metrics were strongly correlated with UE-FMA and UE-FMAp scores and moderately correlated with ARAT scores. Movement duration was very strongly correlated with all TDSM and only moderately correlated with SPARC. IoC was moderately to strongly correlated with all smoothness metrics. Overall results at D30 are presented in Table 5.

**Table 5:** Spearman correlations (r) between metrics at day 30

	UE-FMA	UE-FMAp	ARAT	Duration	IoC
SPARC	0.63^**^	0.66^**^	0.46^**^	-0.58^**^	-0.58^**^
LDLJ	0.66^**^	0.66^**^	0.47^**^	-0.82^**^	-0.62^**^
NARJ	-0.74^**^	-0.73^**^	-0.55^**^	0.89^**^	0.68^**^
nSUB	-0.61^**^	-0.60^**^	-0.40^*^	0.81^**^	0.60^**^

^**^: *p* < 0.01, UE-FMA: upper limb Fugl Meyer score, UE-FMAp: proximal subscore of UE-FMA, ARAT: Action Research Arm Test, IoC: index of curvature, SPARC: spectral arc length metric, LDLJ: log dimensionless jerk, nSUB: number of submovements, NARJ: normalized average rectified jerk

3) Predictive validity.

SPARC at D0 was moderately correlated with UE-FMA, UE-FMAp and ARAT at D30, whereas TDSM at D0 were not correlated with clinical metrics at D30. SPARC at D0 was strongly correlated with IoC at D30, whereas TDSM at D0 were either not correlated (nSUB) or moderately correlated (LDLJ, NARJ) with IoC at D30. Weak (NARJ and nSUB) to moderate (SPARC and LDLJ) correlations were observed between smoothness metrics at D0 and movement duration at D30. Correlations between D0 smoothness metrics and D30 clinical and kinematic metrics are presented in Table 6.

**Table 6:** Spearman correlations (r) between day 0 smoothness metrics and day 30 clinical and kinematic metrics

D30/ D0	UE-FMA	UE-FMAp	ARAT	Duration	IoC
SPARC	0.57^**^	0.54^**^	0.58^**^	-0.55^**^	-0.69^**^
LDLJ	0.21	0.17	0.31	-0.42^*^	-0.40^*^
NARJ	-0.22	-0.18	-0.33	0.39^*^	0.41^*^
nSUB	-0.18	-0.10	-0.26	0.39^*^	0.26

D0: day 0; D30: day 30; ^*^: *p* < 0,05, ^**^: *p* < 0.01, UE-FMA: upper limb Fugl Meyer score, UE-FMAp: proximal subscore of UE-FMA, ARAT: Action Research Arm Test, IoC: index of curvature, SPARC: spectral arc length metric, LDLJ: log dimensionless jerk, nSUB: number of submovements, NARJ: normalized average rectified jerk

Supplementary results for correlations between smoothness metrics are presented in Appendix B.

## Discussion

This study assessed four smoothness metrics in people with moderate to severe motor impairment before and after one month of intensive physical rehabilitation in the subacute phase of stroke. Large clinical and kinematic improvements occurred between D0 and D30. Reliability, responsiveness and construct validity of the smoothness metrics were assessed. Only SPARC and LDLJ had an excellent reliability. Measurement error was lowest for SPARC, followed by LDLJ. Changes in SPARC were correlated with changes in movement duration and more strongly with changes in IoC, while TDSM (NARJ, nSUB and LDLJ) responsiveness was skewed towards movement duration. Most construct validity hypotheses were verified except for TDSM with lower correlations than expected with clinical metrics at D0, ensuing low predictive correlations with clinical metrics at D30.

In the present study, most clinical and kinematic metrics improved from D0 to D30. cMAS and shoulder PROM did not change, but these measures are characterized by uncertain validity and sensitivity to change [43–46]. Movement duration, trajectory length, straightness and smoothness were abnormal at D0, as is generally observed after stroke [16, 47], and all improved significantly by D30 with large effect size. Kinematic outcomes can provide an accurate indication of UE motor recovery after stroke [48]. Participants with high-to-normal UE-FMA still showed deficits in movement kinematic outcomes in a study [39]. UE-FMA and SPARC improved in a longitudinal study of people with mild stroke [23], and is also suggested by our results in people with moderate-to-severe stroke. A recent meta-analysis found that smoothness (measured with nSUB) was the most responsive after stroke among few kinematic outcomes (movement duration, peak velocity, shoulder active range of motion (AROM), control strategy, IoC, elbow AROM and trunk AROM) and that it was as responsive to change as the UE-FMA, indicating that clinical and kinematic measures are complementary and provide a comprehensive and accurate follow-up of motor recovery [18].

### Measurement properties

SPARC and LDLJ displayed an excellent reliability and a low measurement error, while nSUB and NARJ displayed only good and moderate reliabilities respectively and a high measurement error. This result was expected as SPARC and LDLJ were developed to be more reliable than previous metrics [30, 34].

SPARC changes from D0 to D30 were more strongly correlated with changes in movement straightness as assessed by IoC than with changes in movement duration, which can be interpreted as a satisfying responsiveness from a kinematic point of view. This result is complementary to clinical longitudinal validity of SPARC with UE-FMA observed by Saes et al. in 40 individuals followed from week 1 to week 26 after a mild stroke [23]. In contrast, the changes in TDSM were very strongly correlated with the changes in movement duration, but not with changes in movement straightness. The magnitude of change was closer to IoC for the SPARC and closer to movement duration for TDSM, which supports the aforementioned results. This difference in responsiveness suggests a bias of movement duration in the construct measured by TDSM, as was previously found in healthy subjects [34]. The explanation may come from the known noise sensitivity of TDSM [19, 30, 34] as additional noise is mechanically recorded with longer movement duration.

At D0, SPARC more strongly correlated with UE-FMA and ARAT scores than did TDSM. However, at D30, the correlations for SPARC and TDSM with UE-FMA and ARAT scores were of similar strength. Moreover, at D0, SPARC very strongly correlated with movement straightness and moderately with movement duration while TDSM very strongly correlated with movement duration and moderately with movement straightness. Again, differences in correlations were less important at D30. Finally, SPARC values at D0 were more strongly correlated with kinematic and clinical measures at D30 than the TDSM, suggesting stronger predictive validity of SPARC. These differences may be again explained by the known noise-sensitivity ensuing movement duration dependence of TDSM [19, 30, 34], which could have been a more pronounced issue at D0 as the movements were slower and thus may have generated a higher number of signal artefacts.

We found notably stronger correlations between each TDSM than between TDSM and SPARC both at D0 and at D30 (results in Appendix B), suggesting that the TDSM are measuring a very similar construct and that the SPARC is measuring a close but different construct. This is in line with data previously reported for theoretical models and healthy individuals [30, 31, 33]. Smoothness metrics values in the present study differed notably from those in healthy subjects [33] which verified our hypothesis. In particular, the SPARC values during reaching movements of healthy individuals (approximately -1.44 ± 0.02) reported in studies by Engdahl et al. [31], Saes et al. [23] and Bayle et al. [33] differ notably from the values found in the present study (D0: -1.82 and D30: -1.61). In addition, the values for the participants with mild stroke in the study by Saes et al. (week 1: -1.72, week 5: -1.53) [23] differed from both healthy individuals and the participants with a more severe stroke included in the present study. These findings support a discriminant aspect of the construct validity of SPARC.

Finally, the usefulness of adding movement smoothness to the stroke standard assessment is yet to be fully determined even if the addition of kinematic movement quantification has been strongly encouraged by an international consensus [49]. Overall, the measurement properties of smoothness metrics assessed in this clinical study complete the mathematical and simulated results of Mohamed Refai et al. [19] and reinforce the recommendation of the SPARC for the assessment of reaching tasks after stroke.

### Study limitations

Only univariable analyses were conducted owing to the non-normal distribution of the data and the small number of participants. Thus, the correlations, despite their consistency, may be biased by confounding factors. The results may not be generalizable to the people with milder impairments or other types of abnormal movement. The ideal filtering for an optimal noise-to-signal ratio has not been determined for the different smoothness metrics even if we chose the best performing cut-off frequency from a previously published work including tests on filtering and smoothness metrics [31]. Another choice of filter may have improved TDSM performance, especially at D0. High intra-individual variability was observed at D0 for nSUB and NARJ which had higher CoVs than LDLJ and SPARC; thus, assessing more UE movements could have led to steadier results. Recording five trials for participants with more severe impairment could be a pragmatic compromise between data robustness and participant fatigue in future studies, as recently suggested [50].

## Conclusion

The results of this study increase the knowledge of smoothness metrics reliability, responsiveness and construct validity for the assessment of upper limb reach-to-point movements in the subacute phase of stroke. We recommend using SPARC rather than LDLJ to assess the smoothness of reaching movements of uncontrolled duration based on our findings and concordant literature. NARJ and nSUB provide less valid and reliable results in this context. The gathering of validity evidence is an ongoing process, therefore future studies using SPARC or other smoothness metrics in different setups or populations should report their findings concerning measurement properties.

### Electronic supplementary material

Below is the link to the electronic supplementary material.

Supplementary Material 1

Supplementary Material 2

## Data Availability

The dataset supporting the conclusions of this article is included within the article (and its additional files), more details are available on reasonable request.

## Abbreviations

ARAT: Action Research Arm Test
cMAS: Composite modified Ashworth scale
CoV: Coefficient of variation
D0: Day 0 (inclusion in the rehabilitation program)
D30: Day 30 (end of the rehabilitation program)
Δ: D30 value minus D0 value
IoC: Index of curvature
LDLJ: Log dimensionless jerk
NARJ: Normalized average rectified jerk
nSUB: Number of submovements
PROM: Passive range of motion
SPARC: Spectral arc length metric
TDSM: Temporal domain smoothness metrics
UE-FMA: Upper limb Fugl Meyer Assessment
UE-FMAp: Proximal subscore of UE-FMA
