# Supplementary material for: Measurement properties of movement smoothness metrics for upper limb reaching movements in people with moderate to severe subacute stroke
Source: J Neuroeng Rehabil. 2024 May 29;21:90. doi: 10.1186/s12984-024-01382-1 (PMC11134951; doi:10.1186/s12984-024-01382-1)
Supplement: Supplementary file 2 — Supplementary Material 2 [file 12984_2024_1382_MOESM2_ESM.docx]

**Appendix A**

Data base – see Excel file

**Appendix B**

Supplementary Spearman correlations between metrics.

*^*^: p<0,05, ^**^: p<0.01, SPARC: spectral arc length metric, LDLJ: log dimensionless jerk, nSUB: number of submovements, NARJ: normalized average rectified jerk, Δ: change from D0 to D30 (D30-D0 value)*

Correlations between smoothness metrics at D0

|  | SPARC | LDLJ | NARJ | nSUB |
| --- | --- | --- | --- | --- |
| nSUB | -0,57^**^ | -0,88^**^ | 0,87^**^ | 1 |
| NARJ | -0,66^**^ | -0,90^**^ | 1 |  |
| LDLJ | 0,73^**^ | 1 |  |  |
| SPARC | 1 |  |  |  |

Correlations between smoothness metrics at D30

|  | SPARC | LDLJ | NARJ | nSUB |
| --- | --- | --- | --- | --- |
| nSUB | -0,75^**^ | -0,95^**^ | 0,93^**^ | 1 |
| NARJ | -0,74^**^ | -0,94^**^ | 1 |  |
| LDLJ | 0,85^**^ | 1 |  |  |
| SPARC | 1 |  |  |  |

Correlations between smoothness metrics at D0 and at D30

| D30  D0 | SPARC | LDLJ | NARJ | nSUB |
| --- | --- | --- | --- | --- |
| SPARC | 0,65^**^ | 0,60^**^ | -0,61^**^ | -0,52^**^ |
| LDLJ | 0,30 | 0,41^*^ | -0,41^*^ | -0,35 |
| NARJ | -0,25 | -0,37^*^ | ,38^*^ | 0,27 |
| nSUB | -0,29 | -0,37^*^ | ,36^*^ | 0,30 |

Correlations between smoothness metrics at D0 and clinical change from D0 to D30 (Δ)

| D0  Δ | SPARC | LDLJ | NARJ | nSUB |
| --- | --- | --- | --- | --- |
| ΔUE-FMA | 0,45* | 0,11 | -0,13 | -0,11 |
| ΔARAT | 0,37* | 0,14 | -0,15 | -0,16 |
